# HOW CARE WORKERS’ TURNOVER INTENTION IS ASSOCIATED WITH WORKPLACE VIOLENCE?: THE ROLE OF STRESS AND JOB SATISFACTION

**DOI:** 10.1093/geroni/igad104.0774

**Published:** 2023-12-21

**Authors:** Sung Hyun Ko, Yeonjung Lee

**Affiliations:** Chung-Ang University, Seoul, Republic of Korea; Chung-Ang University, Seoul, Republic of Korea

## Abstract

This study examines the impact of workplace violence experienced by long-term care workers from the care recipients on their turnover intention and the mediating effect of work-related stress and job satisfaction in the relationship between workplace violence and turnover intention. Compared to the unfair experiences of abuses experienced by care recipients, care workers’ experiences have been relatively under-explored. Adopting the affective events theory as a theoretical framework and using the Korean National Long-Term Care Survey in 2019, the current analysis focuses on 2,427 care workers, excluding social workers, nurses, and occupational/physical therapists. The results of structural equations modeling and effect decomposition show a mediating effect of job satisfaction, indicating that the experience of workplace violence decreased the job satisfaction of care workers, resulting in increased turnover intention. On the other hand, workplace violence did not have a statistically significant indirect effect on turnover intention through work-related stress. Findings provide several implications for developing an inclusive and supportive policy and practice for care workers as well as the long-term care system in general in South Korea. Support programs and policies can be targeted toward the care workers who have experienced workplace violence and are less satisfied with their job. These can be done not only by highlighting the importance of training and education for care workers to help prevent workplace violence but also by providing a safe work environment, an appropriate compensation system, and the fair opportunities for promotion.

